# Lanthanum and Neodymium Doped Barium Ferrite-TiO_2_/MCNTs/poly(3-methyl thiophene) Composites with Nest Structures: Preparation, Characterization and Electromagnetic Microwave Absorption Properties

**DOI:** 10.1038/srep20496

**Published:** 2016-02-09

**Authors:** Jie Zhao, Jian Yu, Yu Xie, Zhanggao Le, Xiaowei Hong, Suqin Ci, Junhong Chen, Xiaoyan Qing, Weijie Xie, Zhenhai Wen

**Affiliations:** 1 College of Environment and Chemical Engineering, Nanchang Hangkong University, Nanchang, 330063, PR China; 2 Department of Mechanical Engineering, University of Wisconsin-Milwaukee, Milwaukee, 53211, USA; 3 Fujian Institute of Research on the Structure of Matter, Chinese Academy of Sciences, Fuzhou, Fujian, 350002, PR China; 4 Department of Applied Chemistry, East China Institute of Technology, Nanchang, 330013, PR China

## Abstract

We report herein the synthesis of a novel nest structured electromagnetic composite through *in-situ* chemical polymerization of 3-methyl thiophene (3MT) in the presence of the BaFe_11.92_(LaNd)_0.04_O_19_-TiO_2_ (BFTO) nanoparticles and MCNTs. As an absorbing material, the BFTO/MCNTs/P3MT/wax composites were prepared at various loadings of BFTO/MCNTs/P3MT (0.2:0.10:1.0 ~ 0.2:0.30:1.0), and they exhibited strong microwave absorption properties in the range of 1.0–18 GHz. When the loading of BFTO/MCNTs/P3MT is 0.2:0.30:1.0, the composite has a strongest absorbing peak at 11.04 GHz, and achieves a maximum absorbing value of −21.56 dB. The absorbing peak position moves to higher frequencies with the increase of MCNTs content. The mechanism for microwave absorption of these composites has been explained in detail.

In recent years, microwave absorbing materials have received great attention because their potential application in the field of electromagnetic shielding. Nevertheless, the microwave absorbing materials still have many shortcomings, such as skin effect, easy oxidation, poor heat stability and narrow absorption frequency^1^,^2^,^3^,^4^,^5^,^6^,^7^,^8^. Therefore, novel microwave absorbing materials with high-performance are highly desirable for the scientific and technological development and the microwave absorption field. Unfortunately, one single material normally cannot meet the requirements of strong and wide bandwidth absorption. Consequently, the composites are promising to become the future microwave absorbing material because they can hold the advantages of each building block unit while making up for each other’s shortcomings. For these reasons, the composites will play an important role in breaking the “bottleneck” for developing high-performance microwave absorbing materials.

Recently, extensive studies have shown doping rare earth in some conventional microwave absorbing material can significantly promote the ferrite magnetic anisotropy field, improve coercivity and enhance ferrite electromagnetic properties, thereby increasing the magnetic hysteresis loss in the alternating electromagnetic field^9^. Beside this, the doped rare earth ions with larger radiuses can induce the lattice distortion with improving the dielectric loss^10^. In addition, it was widely reported that conductive polymers of polythiophene (PTh) and its derivatives have good dielectric properties and strong dielectric loss capacity^11^,^12^,^13^; such property can compensate for the insufficient dielectric loss of ferrite and improve the ferrite microwave absorbing properties. Furthermore, multiwall carbon nanotubes (MCNTs) also possess good dielectric loss ability with advantages of small size, quantum effects, microwave absorption properties and so on^14^,^15^,^16^,^17^,^18^,^19^,^20^. It is envisaged that MCNTs conjugating with PTh through π-π can improve the electrical conductivity and enhance the dielectric loss. In this way, the introduction of the MCNTs may be beneficial to adjusting the composite’s microwave absorption property. As is well known, barium ferrite possesses chemical stability, high permeability and relatively smaller dielectric constant; these properties benefit for the impedance matching and microwave absorption^9^. However, the barium ferrite has defects of the high density and narrow microwave absorption bandwidth.

Bearing these points in mind, we herein reported the integration of barium ferrite, rare earth metal, TiO_2_, MCNTs and poly(3-methyl thiophene) (P3MT) in a composites system, i.e., BaFe_11.92_(LaNd)_0.04_O_19_-TiO_2_/MCNTs/P3MT (BFTO/MCNTs/P3MT), targeting to develop a promising microwave absorption material.

## Experiment

### Materials

MCNTs were purchased from Beijing DK nano technology Co. Ltd. 3-methyl thiophene (3MT, C_5_H_6_S), HCl, (NH_4_)_2_S_2_O_8_ (APS), Ba(NO_3_)_2_, La(NO_3_)_3_·9H_2_O, Nd(NO_3_)_3_·5H_2_O, Fe(NO_3_)_3_·9H_2_O, citric acid (C_6_H_8_O_7_·H_2_O), ethylene glycol (C_2_H_6_O_2_), tetrabutyl titanate (C_16_H_36_O_4_Ti) and NH_3_·H_2_O are all analytical reagent grade.

### Purification of MCNTs

MCNTs were added in a concentrated nitric acid and refluxed at 90 °C for 5 h. After that, the suspension was filtered. Then, the black solids were firstly washed with 0.1 mol/L HCl and then with deionized water, respectively. Finally, the purified MCNTs powder were obtained under vacuum at 50 °C for 24 h.

### Preparation of the BaFe_11.92_(LaNd)_0.04_O_19_-TiO_2_ (BFTO) composites

The BFTO composites were prepared according to our previous reports^20^,^21^. The BaFe_11.92_(LaNd)_0.04_O_19_ wet gel and the TiO_2_ gel were mixed in the mass ratio of 3:5.

### Preparation of the BFTO/MCNTs/P3MT composites

The BFTO/MCNTs/P3MT composites were prepared by the method that we reported^20^. The mass ratio of BFTO, MCNTs, and 3MT monomers were 0.2:0.10:1.0, 0.2:0.15:1.0, 0.2:0.20:1.0, 0.2:0.25:1.0, and 0.2:0.30:1.0, respectively. For convenience, the composite is named according to the mass ratio of each unit. For instance, when the composite has a mass ratio of 0.2:0.10:1.0 for BFTO, MCNTs, and 3MT monomers, the composites was named as BFTO/MCNTs/P3MT (0.2:0.10:1.0).

### Characterization and electromagnetic properties measurement

The morphology, structure and properties of samples were characterized by various techniques. Fourier transform infrared (FTIR) spectra were carried out using Nicolet 5700 FTIR with a KBr method. X-ray diffraction (XRD) patterns of the samples were characterized using a Philps-pw3040/60 diffractometer with Cu Kα radiation (λ = 0.15418 nm). Differential thermal analysis-thermo gravimetry (DTA-TG) analysis was performed at a heating rate of 10 °C in nitrogen on SDTQ 600. The morphology and particle sizes of the samples were characterized by a Hitachi H-800 scanning electron microscope (SEM) and a JEOL JEM-1200EXII transmission electron microscope (TEM). Vector Network Analyzer (HP-8722ES) was used to get S-parameters for the samples of composites in the range of 1–18 GHz at room temperature. The values of complex permittivity (ε) and permeability (μ) of the composite materials were calculated from the measured values of S-parameters. The reflection loss of the single layer sample was calculated using the measured electromagnetic parameters.

## Results and Discussion

### Polymerization

**Figure 1:**
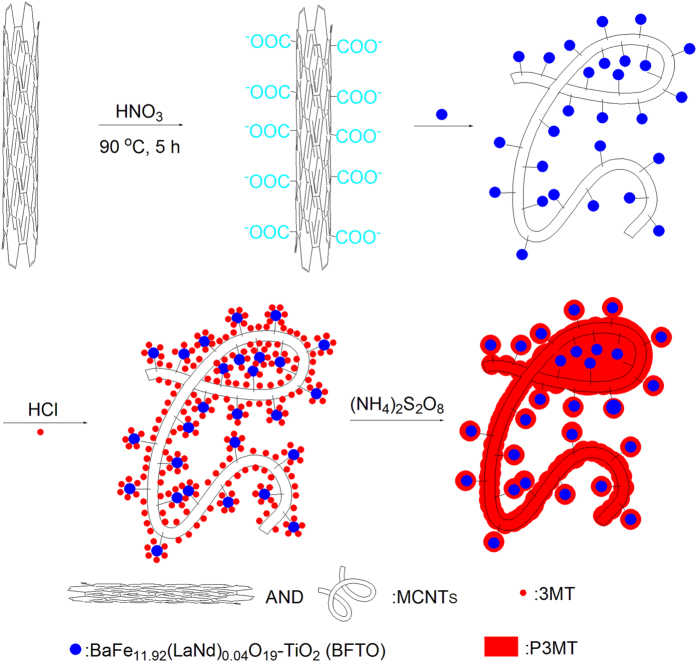
Schematic of the BFTO/MCNTs/P3MT composites.

Figure 1 illustrates the preparation process of the BFTO/MCNTs/P3MT composites. Firstly, MCNTs are refluxed in concentrated HNO_3_ solution at 90 °C for 5 h to remove the residues of metal catalysts; such treatment could produce a large number of carboxylic groups on the surface of MCNTs that benefit for absorbing the BFTO nanoparticles. Then, Cl^−^ is absorbed onto the surface of BFTO nanoparticles by the electrostatic attraction. 3MT monomers are attracted by Cl^−^ through the electrostatic effect and uniformly distributed over the surface of MCNTs through п-п stacking. Finally, the target products with a nest structures are obtained by *in-situ* chemical polymerization of 3MT with the (NH_4_)_2_S_2_O_8_ as an initiator.

### XRD analysis

**Figure 2:**
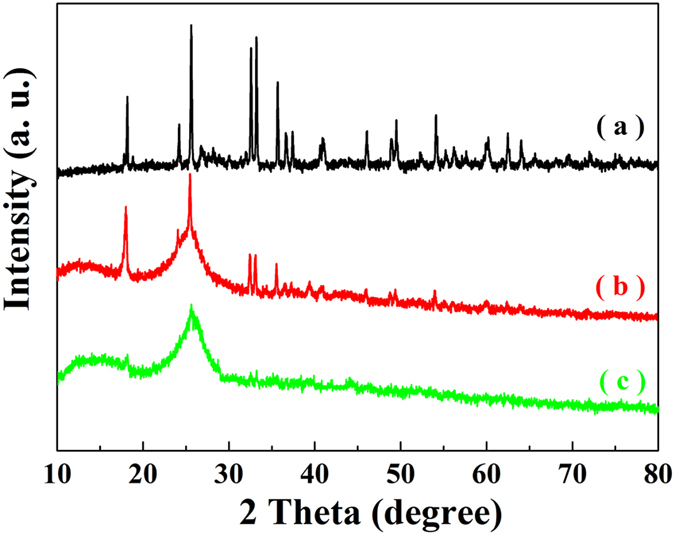
XRD patterns of (**a**) BFTO, (**b**) BFTO/MCNTs/P3MT, (**c**) P3MT.

The diffraction patterns of the samples are presented in Fig. 2. Figure 2a shows the characteristic diffraction peaks of the BFTO. The peaks at 2θ = 25.2°, 33.6° and 36.1° are ascribed to the characteristic diffraction peaks of BaFe_11.92_(LaNd)_0.04_O_19_^20^,^21^. And the peaks at 2θ = 17.9°, 25.5° and 33.6° are attributed to the characteristic diffraction peaks of TiO_2_^22^. The typical XRD pattern of P3MT (Fig. 2c) presents two broad diffraction peaks centered at 2θ = 14.5° and 26.5° with shift slightly^23^, which can be ascribed to the intermolecular π-π stacking emerges. Figure 2(b) shows the XRD pattern of the BFTO/MCNTs/P3MT composites, which contains the characteristic diffraction peaks of BFTO, MCNTs and P3MT. It should be noted that the intensity of characteristic diffraction peaks of P3MT in the composites is weaker compared to the pristine P3MT, which can be attributed to the interactions among BFTO, MCNTs and P3MT and the nest structures of the composites.

### FTIR analysis

**Figure 3:**
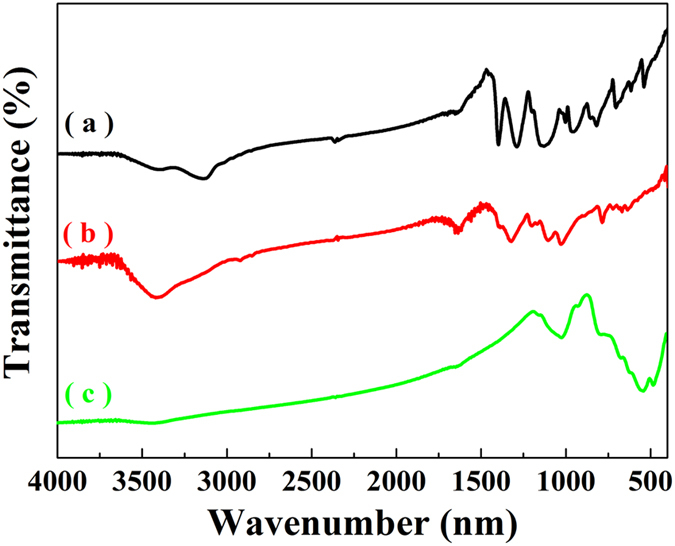
FTIR spectra of P3MT (**a**), BFTO/MCNTs/P3MT (**b**) and BFTO (**c**).

Figure 3 shows the FTIR spectra of BFTO, BFTO/MCNTs/P3MT and P3MT, respectively. For P3MT (Fig. 3a), two peaks in the range of 2750–3000 cm^−1^ are attributed to the characteristic C-H stretching vibrations and the peak at 1640 cm^−1^ is assigned to C = C stretching vibrations. The peak at 784 cm^−1^ is assigned to the C-H out-of-plane vibrations of the 2, 5-substituted thiophene ring created by the polymerization of thiophene monomers. The peak at around 692 cm^−1^ denotes the C-S stretch in the thiophene ring^24^. For BFTO (Fig. 3c), the peaks at 596 and 440 cm^−1^ are attributed to the characteristic Fe-O and Ti-O stretching vibration band, respectively^22^. Figure 3b shows the BFTO/MCNTs/P3MT FTIR spectra, which is almost identical to that of P3MT. However, to some extent, the spectra of the P3MT in the composites appear slightly blue shift. In addition, the intensity of the peak at 696.5 cm^−1^ becomes weaker compared with Fig. 3c. The peaks at 1112.5 cm^−1^ and 1627.4 cm^−1^ are attributed to the MCNTs’ characteristic absorption peaks with slightly blue shift compared to the literature^25^, suggesting that the BFTO and MCNTs are well coated by P3MT chains. Because there exists some interactions among them in the composites, which decreases the electron density and reduces the atomic force constant. These above results confirm that composites are composed of the P3MT, BFTO and MCNTs.

### DTA-TG analysis

**Figure 4:**
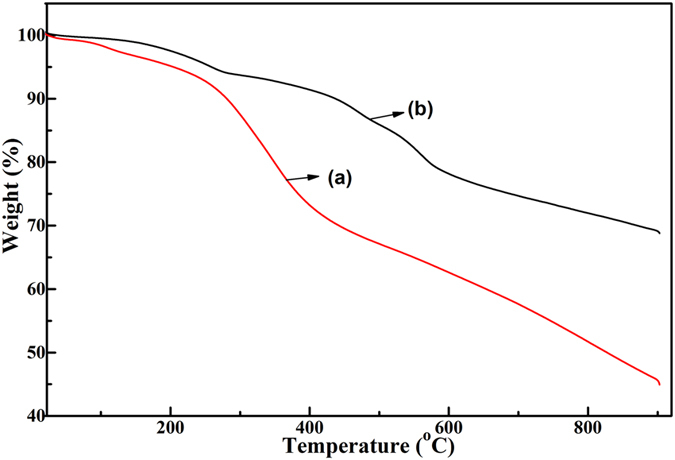
DTA-TG analysis of (**a**) P3MT and (**b**) BFTO/MCNTs/P3MT.

DTA-TG analysis of BFTO/MCNTs/P3MT and P3MT and are shown in Fig. 4. The weight loss of the two samples can be divided into three stages. For the P3MT (Fig. 4a), the first stage is assigned to the loss of water and other volatiles at lower temperature (lower than 110 °C). The second stage above 197 °C can be attributed to the thermal degradation of the P3MT chains and volatilization of the oligomer. The third stage of P3MT is starting at 500 °C. The TG curve (Fig. 4b) indicates that the decomposition temperature of the BFTO/MCNTs/P3MT composites is about at 300 °C, higher than that of pure P3MT. The third weight loss of the composites starts at 610 °C, indicating that the stability of the composites is better than that of P3MT. The improved stability may be resulted by the interactions among the P3MT, BFTO and MCNTs or the nest structures of the BFTO/MCNTs/P3MT composites.

### Morphology analysis

**Figure 5:**
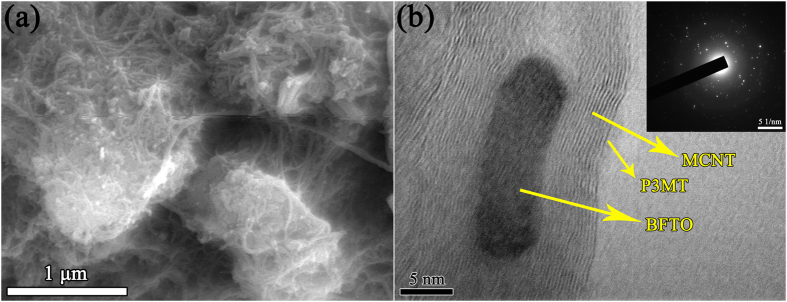
SEM (**a**) and TEM (**b**) images of BFTO/MCNTs/P3MT.

The SEM images of P3MT and BFTO/MCNTs/P3MT composites are shown in Fig. 5a. It can be seen that lots of bending long tubes agglomerated densely were coated by P3MT. And the composites have an irregular and similar nest structure. The introduction of hydrochloride can increase the polarity of P3MT, resulting in the increase of intermolecular force.

Figure 5b shows the TEM images of BFTO/MCNTs/P3MT. There is a typical tube morphology of MCNTs which are conjugated with BFTO nanoparticles. In addition, both MCNTs and BFTO nanoparticles are coated by P3MT. Electronic diffraction pattern indicates that the black core is BFTO, because only BFTO composite is crystal material in the BFTO/MCNTs/P3MT composites. BFTO is absorbed onto the surface of the MCNTs. These results confirm that the composites are composed of polycrystalline BFTO, MCNTs and P3MT, being in accordance with the results of FIRT and XRD analysis.

### Electromagnetic parameter analysis

**Figure 6:**
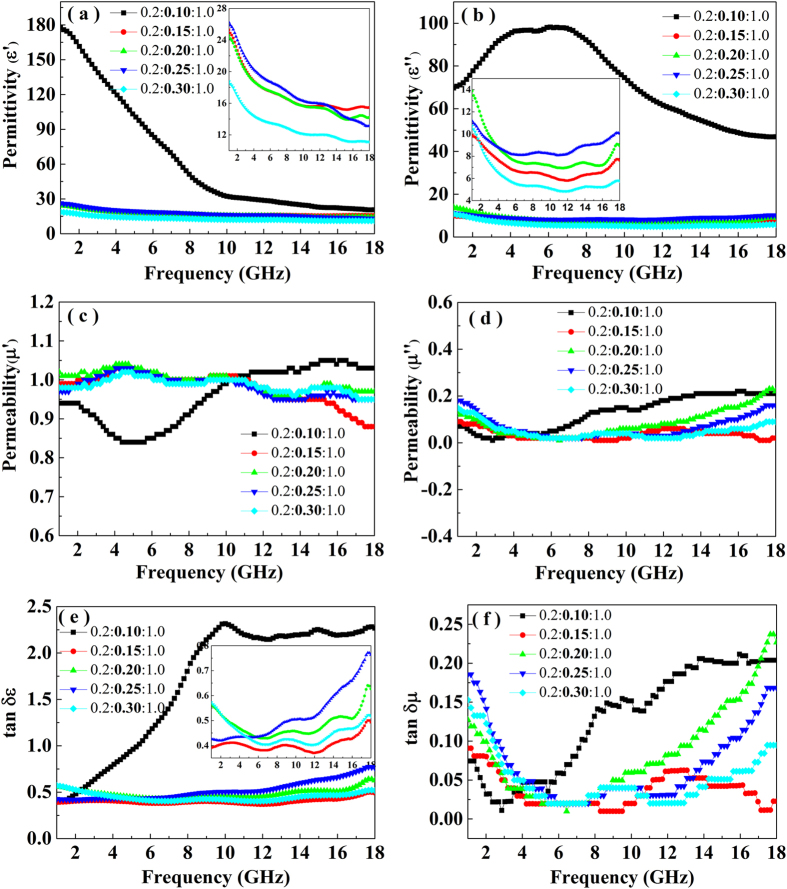
The ε′ (**a**), ε″ (**b**), μ′ (**c**), μ″ (**d**), tgδ_ε_ (**e**) and tgδ_μ_ (**f**) of the BFTO/MCNTs/P3MT/wax composites with different BFTO/MCNTs/P3MT loading.

To investigate the electromagnetic wave absorption properties of the BFTO/MCNTs/P3MT, various contents of the as-prepared powder was mixed with wax (the mass ratio is 7:3) to form the BFTO/MCNTs/P3MT/wax composites by a hot press process. Figure 6a–d shows the real and imaginary parts of the complex permittivity and permeability measured for the composites with different mass ratio of the BFTO/MCNTs/P3MT in the range of 1.0–18 GHz. As shown in Fig. 6a,b, the real (ε′) and imaginary (ε″) parts of the permittivity obviously reduce first, then slightly increase and then decrease with the increase of MCNTs content. The ε′ value of above the BFTO/MCNTs/P3MT/wax decreases with increasing frequency in the range of 1.0–18 GHz. However, the changes of ε″ values were very complicated with the different contents of MCNTs in the composites. The ε″ values of the composites (with a loading of the BFTO/MCNTs/P3MT (0.2:0.15:1.0 ~ 0.2:0.30:1.0) decrease first, then increase slightly. However, for the loading of BFTO/MCNTs/P3MT (0.2:0.10:1.0), the ε″ increases first, then decreases. The ε′ and ε″ values of the composite (with a loading of the BFTO/MCNTs/P3MT (0.2:0.30:1.0)) decrease from 18.76 (maximum) to 11.15 (minimum) and 10.75 (maximum) to 4.84 (minimum) respectively in the frequency range of 1.0–18 GHz. However, when the BFTO/MCNTs/P3MT is 0.2:0.10:1.0, the ε′ and ε″ values decreases from 177.78 (maximum) to 20.70 (minimum) and 98.17 (maximum) to 46.91 (minimum) respectively. The ε′ (177.78) and ε″ (98.17) values are higher than those of other reports^26^, it indicates that the introduction of MCNTs into the BFTO/MCNTs/P3MT composite can greatly enhance the dielectric constant. The enhanced dielectric constant can be attributed to the excellent dielectric properties of MCNTs and the synergistic effects between different components in the composites, which is consistent with the results of other studies for MCNTs^27^ and Fe_3_O_4_-MCNTs^28^. With the increase of MCNTs content, the ε′ and ε″ values of the other composites were lower than those of the composite with a loading of 0.2:0.10:1.0. This can be explained by the percolation theory^29^. It is well-known that percolation behaviour corresponds to a phase transition from a conducting state to an insulator state for the composites around the percolation points. The lower values of the ε′ and ε″ for those composites (with a loading of the BFTO/MCNTs/P3MT (0.2:0.15:1.0 ~ 0.2:0.30:1.0) can also keep relatively high level (26.22 ~ 10.75; 13.88 ~ 4.84) than other reports^30^. As we known, the real part of permittivity is an expression of the polarizability of a material, which consists of dipolar polarization and electric polarization at microwave^31^. The relatively high real part of the permittivity can be explained by the fact that the conducting MCNTs may increase the electric polarization of the sample compared to that of the Fe_3_O_4_-Polyanine^32^. The dielectric loss angle tangent (tanδ_ε_ = ε″/ε′) was also calculated as shown in Fig. 6e. The values of dielectric loss tangent for the composites (with a loading of the BFTO/MCNTs/P3MT (0.2:0.15:1.0 ~ 0.2:0.30:1.0) fluctuate between 0.37 and 0.77. When the loading of BFTO/MCNTs/P3MT is 0.2:0.10:1.0, the value of dielectric loss tangent is as high as 2.32. According to the electromagnetic theory, such high dielectric loss results from the naturally physical properties and unique structures of the composites. First, the existence of residual defects in MCNTs and dangling band atoms and unsaturated coordination on the surface of the BFTO/MCNTs/P3MT hybrids is in favor of the electromagnetic energy absorption^33^,^34^. Second, the interfaces polarizations of BFTO/MCNTs, BFTO/P3MT, MCNTs/P3MT, BFTO/wax, MCNTs/wax and P3MT/wax account for relaxation process with respect to a changing electric field in a dielectric medium^35^.

Figure 6c,d show the real part (μ′) and imaginary part (μ″) of the permeability of the composites. The μ′ values of all the composites fluctuate between 0.84 and 1.05 in the frequency of 1.0–18 GHz. And the μ″ values of permeability, as shown in Fig. 6d, mainly fluctuate between 0.88 and 1.05 in the frequency of 1.0–18 GHz. Compared with higher complex permittivity, the complex permeability of the BFTO/MCNTs/P3MT/wax is very lower. It indicates that the magnetic loss contribution from BFTO/MCNTs/P3MT to microwave absorption is minor. The magnetic loss angle tangent (tanδ_μ_ = μ″/μ′) was been calculated and shown in Fig. 6f. The values of the magnetic tangent loss show very small fluctuation between 0.01 and 0.24. In addition, for all the composites with different loading of BFTO/MCNTs/P3MT, the dielectric loss tangent (tanδ_ε_) is larger than the magnetic loss tangent (tanδ_μ_). Thus, for every composites, the dielectric loss is the main contribution for microwave absorption.

### Microwave absorption properties

To reveal the microwave absorption properties of the BFTO/MCNTs/P3MT/wax composites, the reflection loss (R_L_) values were calculated according to the transmission line theory by the following equations^36^,^37^


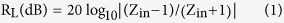



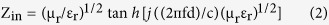


where ε_r_ and μ_r_ are the complex permittivity and permeability, respectively; R_L_ is a ratio of reflected power to incident power in dB, Z_in_ is the input impedance of absorber, d is the thickness of the absorber, c is the velocity of light, f is the frequency of microwave.

**Figure 7:**
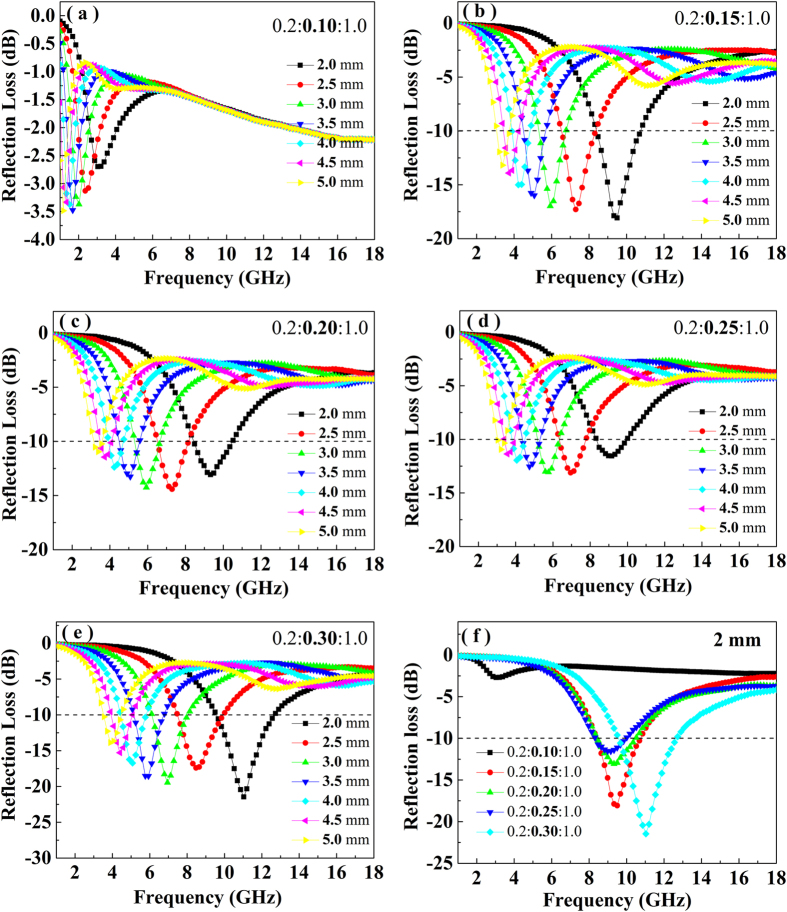
Microwave reflection loss (R_L_) curves of the BFTO/MCNTs/P3MT composites of (**a**) 0.2:0.10:1.0, (**b**) 0.2:0.15:1.0, (**c**) 0.2:0.20:1.0, (**d**) 0.2:0.25:1.0, (**e**) 0.2:0.30:1.0 with different thicknesses in the frequency range of 1.0–18.0 GHz; (**f**) Microwave reflection loss (R_L_) curves of the BFTO/MCNTs/P3MT composites with a thickness of 2.0 mm in the frequency range of 1.0–18.0 GHz.

Figure 7a–e show the theoretical R_L_ of the BFTO/MCNTs/P3MT/wax composites with different thickness (2.0–5.0 mm) in the range of 1.0–18 GHz with the BFTO/MCNTs/P3MT loading of 0.2:0.10:1.0 ~ 0.2:0.30:1.0. It can be seen that the microwave absorption properties and the R_L_ peak can be tuned by controlling the thickness of the absorbers. For the BFTO/MCNTs/P3MT/wax composites with a loading of 0.2:0.30:1.0, there is a stronger peak (−21.56 dB at 11.04 GHz), when the absorbers with a thickness of 2.0 mm. The peaks of other composites with different BFTO/MCNTs/P3MT loading are at frequency of 1.16 GHz (−3.49 dB, 0.2:0.10:1.0), 9.51 GHz (−18.11 dB, 0.2:0.15:1.0), 7.28 GHz (−18.11 dB, 0.2:0.20:1.0) and 6.98 GHz (−13.17 dB, 0.2:0.25:1.0), respectively. Compared with Fig. 6, we can find that the lower concentration of MCNTs in BFTO/MCNTs/P3MT exhibits higher dielectric loss and results in a worse reflection. When the MCNTs content ratio increase from 0.10–0.30, the dielectric loss of composites decreases inversely and possesses better reflection, which may be explained by the percolation theory^29^. The percolation behavior of composites corresponds to a phase transition from an conducting state to an insulator state around the percolation point. In our BFTO/MCNTs/P3MT/wax composites with 0.2:0.10:1.0, the MCNTs network enhances the electrical conductivity of the composite and this leads to a high leakage current, which may cause damage to the microwave absorption of materials^38^. In addition, impedance match characteristic is an important concept for the microwave absorption. High permittivity of absorber is harmful to the impedance match and results in weak absorption^33^.

Figure 7f shows the theoretical R_L_ of the composites with different BFTO/MCNTs/P3MT loading in the frequency range of 1.0–18 GHz at a thickness of 2.0 mm. It can be seen that the loading of BFTO/MCNTs/P3MT have a great influence on the microwave absorbing properties and the minimum R_L_ corresponding to the maximum absorptions gradually appeared in different frequency shifts toward to higher frequency with the increase of MCNTs contents. When the loading of the BFTO/MCNTs/P3MT is 0.1:0.30:1.0, the minimum R_L_ can be achieved to −21.56 dB at 11.04 GHz, and the bandwidth of R_L_ less than −10 dB can reach up to 3.25 GHz (from 9.50–12.75 GHz). This relatively winder bandwidth may be ascribed to the unique interfaces among BFTO, MCNTs and P3MT, as well as the excellent dielectric properties of MCNTs and P3MT. In addition, the inorganic/organic interfaces between BFTO/MCNTs/P3MT and wax, the synergistic effect between different components in the BFTO/MCNTs/P3MT/wax composites may also be important factors for enhanced microwave absorption performance. It can be obviously observed that the reflectivity peak position moves to higher frequency and the microwave absorption property becomes stronger with the increase of the MCNTs contents loading. These results indicate that the absorption peak positions and frequency ranges (minimum R_L_ less than −10 dB) can be manipulated easily by adjusting the MCNTs concentrations in the BFTO/MCNTs/P3MT, and thus a broadband absorption can be designed using a multilayered absorbing structure.

## Conclusion

The BFTO/MCNTs/P3MT composites have been prepared by *in situ* polymerization of P3MT in the presence of BFTO and MCNTs. The electromagnetic and microwave absorption properties of the BFTO/MCNTs/P3MT/wax composites with different MCNTs loading have been investigated. With different MCNTs content loading, there is a percolation phenomenon for dielectric loss. The higher MCNTs concentration leads to a lower dielectric loss. When the BFTO/MCNTs/P3MT is 0.2:0.30:1.0, the composite shows the best microwave absorption with −21.56 dB at 11.04 GHz. For all the composites, the main contribution for the microwave absorption comes from the dielectric loss rather than the magnetic loss. Considering the absorption peak could be easily adjusted by changing the MCNTs concentration, these composite materials possess a great potential application for broad bandwidth microwave absorption.

## Additional Information

**How to cite this article**: Zhao, J. *et al.* Lanthanum and Neodymium Doped Barium Ferrite-TiO_2_/MWCNTs/poly(3-methyl thiophene) Composites with Nest Structures: Preparation, Characterization and Electromagnetic Microwave Absorption Properties. *Sci. Rep.* **6**, 20496; doi: 10.1038/srep20496 (2016).
